# Assessment of muscle quality by phase angle and body physique in nonathlete students and trained/developmental athletes

**DOI:** 10.14814/phy2.70412

**Published:** 2025-06-11

**Authors:** Kazushige Oshita, Akihisa Hikita, Ryota Myotsuzono, Satoki Murai

**Affiliations:** ^1^ Department of Human Information Engineering Okayama Prefectural University Soja Japan; ^2^ Department of Sport Science Kyushu Kyoritsu University Kitakyushu Japan

**Keywords:** bioelectrical impedance analysis, body mass index, fat‐free mass, lean body mass, muscle mass

## Abstract

Phase angle (PhA), measured using bioelectrical impedance analysis, has recently gained attention as an indicator of muscle quality. This study investigates the relationship between PhA and body mass index (BMI) in nonathletic students and trained/developmental athletes. Seventy‐one male and 83 female students participated in the Normal group, while 159 male and 95 female students participated in the Sports group. Each group was further divided into two subgroups: those with a BMI higher (L‐Normal and L‐Sports) and lower (S‐Normal and S‐Sports) than the median BMI. Although fat‐free mass (FFM) did not differ significantly between the S‐Sports and L‐Normal groups, PhA was significantly higher in the S‐Sports group with a large effect size. While FFM was significantly higher in the L‐Sports group than in the S‐Sports group, PhA did not differ significantly with a small effect size. PhA and BMI showed no significant relationship in the Sports group, whereas a positive correlation was observed in the Normal group. These results suggest that PhA can be used to assess differences in competition and activity levels that are not represented by BMI or FFM. Furthermore, although PhA is related to BMI in the nonathletic populations, this relationship is not observed in trained/developmental‐level athletes.

## 1 INTRODUCTION

Sports science professionals seek to understand how various body components contribute to performance enhancement, injury prevention, and athletic health monitoring (Silva, 2019). Bioelectrical impedance analysis (BIA) is the most commonly used method for measuring body composition due to its relatively low cost, ease of use, and portability (Ellis, 2000). BIA measures impedance (Z) by applying a weak alternating current (AC) to the body. The resistance of tissues to AC is directly related to their fluid content: highly hydrated fat‐free mass (FFM) is a good electrical conductor, whereas poorly hydrated adipose tissue is a good electrical insulator (Bracco et al., 1996). Although Z includes the reactance (Xc) and resistance (R), the phase angle (PhA) calculated from Xc and R is considered an indicator of cell health, with higher values reflecting greater cellularity, cell membrane integrity, and better cell function (Norman et al., 2012). Furthermore, PhA has the advantage of being estimated directly from raw bioelectrical measurements, without requiring conversion equations (Campa & Coratella, 2021).

In addition to the assessment of body composition using BIA, the analysis of resistance characteristics, including PhA, has recently gained attention in sports science (Campa et al., 2021). Reviews of athletic performance suggest that changes in PhA are associated with physical adaptations following training or nutritional interventions, and PhA has been positively correlated with sport‐specific muscle strength and power test outcomes (Campa et al., 2022). Additionally, as PhA increases with resistance training and decreases with detraining (Lukaski & Raymond‐Pope, 2021), it may serve as an indicator of strength and power training effectiveness. PhA is also directly related to aerobic fitness across different age groups, sexes, and health statuses (Custódio Martins et al., 2022), emphasizing its relevance in monitoring exercise effects across populations (Annunziata et al., 2024). The PhA of a healthy individual varies between 5° and 7°, whereas in athletes, it may reach values of 8.5° (Cancello et al., 2023). Reference values for PhA for different sporting events have also been reported (Campa et al., 2022). However, despite increasing research interest in PhA, heterogeneous results in athletes have been observed (Cirillo et al., 2023).

Body weight, body mass index (BMI), and FFM are positively associated with PhA in athletes (Di Vincenzo et al., 2019), and muscle strengthening results in a greater increase in PhA than endurance training (Marra et al., 2021). Consequently, PhA is expected to be higher in athletes with larger body physiques, such as those in explosive sports, compared to athletes with smaller physiques, such as those in endurance sports. Indeed, endurance athletes exhibit lower PhA than those in velocity/power sports, although the magnitude of this difference is reportedly small (Campa et al., 2022). A study on PhA among elite athletes (aged 18–35 years), including professional cyclists and national water polo players, and healthy nonathlete controls found significantly higher PhA in athletes than in controls, but no significant differences among sports (Di Vincenzo et al., 2019). Similarly, a study providing reference values of PhA for 19–22 disciplines in national athletes aged 16 years or older reported 95% confidence intervals for the 50th percentile values range from 7.6° to 7.7° and 6.7° to 7.0° for males and females (Campa et al., 2022), indicating minimal differences across sports. Thus, while PhA is expected to be higher in athletes with larger physiques, no large differences may be observed among sports disciplines.

A possible explanation for these findings is the variation in athletic and activity levels among athletes. While the aforementioned studies describe elite or national athletes, their exact classification remains unclear. Due to the lack of well‐defined classification criteria, such as elite or trained status, within exercise science and physiology, McKay et al. (2022). recently proposed a tier‐based classification system to standardize terminology in sports science literature. According to their classification (McKay et al., 2022), individuals in tier 2 (trained/developing) are expected to have a larger physique (particularly FFM) and higher PhA than those in tier 0 or 1 (sedentary or recreationally active). However, whether PhA differs according to body physique or muscle mass within the same tier of classification remains unknown. Based on a previous research showing a correlation between BMI and PhA, athletes with larger physiques should exhibit higher PhA values even within the same tier category. In contrast, another study found no PhA differences across sports, suggesting that PhA does not differ between athletes with larger and smaller physiques.

In addition, age range should be considered in these relationships of PhA. For instance, PhA in the general population is higher in individuals in their 20s compared to those aged 15–19 years (Oshita et al., 2025). Furthermore, sex differences in PhA increase during adolescence, with adult PhA values consistently higher in males than in females (Marra et al., 2021). Therefore, in athletes aged 16–35 years, age may influence PhA. Consequently, to better understand the relationship between PhA and athletic or activity levels, investigations should focus on specific age groups, such as high school or university students. This study examines the relationship between body physique or composition and PhA in university students classified at tiers 0–1 and 2 based on the classification by McKay et al. (2022).

## 2 MATERIALS AND METHODS

### 2.1 Participants and their classification

The participants were 408 university students in Japan (18–22 years old; 230 males, 178 females). Prior to participating, all students were informed of the purpose and methods of the study, both orally and in writing. Only those who provided written consent were included in the measurements described below. This study was reviewed and approved by the Research Ethics Committee of Kyushu Kyoritsu University (approval number: 2022–08) and was conducted in accordance with the ethical principles of the Declaration of Helsinki.

**FIGURE 1:**
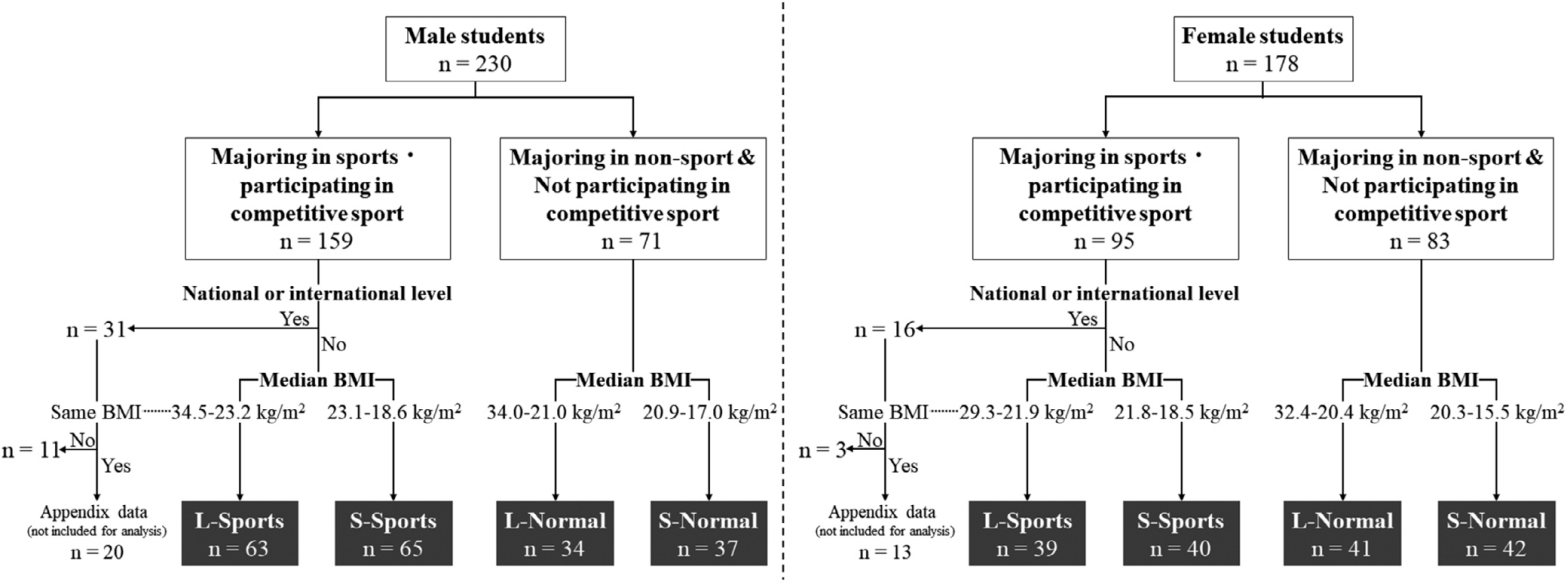
Flow diagram outlining participant selection for this study.

The participants were divided into two groups: university students majoring in sports and participating in competitive sports (Sports group) and non‐sports majors (e.g., information technology and nutrition) who did not participate in competitive sports (Normal group) (Figure 1). The Sports group excluded students who met the performance standards for national or international competition (≥ tier 3) (McKay et al., 2022). Each group was further divided based on median BMI: 23.1 and 21.8 kg/m^2^ for males and females in the Sports group, and 20.9 and 20.3 kg/m^2^ for males and females in the Normal group. Within the Sports group, participants with a higher BMI were classified as the L‐Sports) group (63 males, 39 females), while those with a lower BMI were classified as the small‐Sports (S‐Sports) group (65 males, 40 females). Similarly, in the Normal group, participants with a higher BMI were categorized as the large‐Normal (L‐Normal) group (34 males, 41 females), and those with a lower BMI were classified as the small‐Normal (S‐Normal) group (37 males, 42 females). Among the excluded students with a higher level of competition, those with the same BMI range as the L‐Sports group were selected. Due to the small number of participants in this group, their data are presented in the Appendix.

### 2.2 Body composition and PhA

Body weight and composition were measured using a standing 8‐electrode, multifrequency BIA body composition analyzer (MC‐780A‐N, Tanita, Japan). Body composition was estimated from impedance values at three different AC (5 kHz, 50 kHz, and 250 kHz) with 90 μA or less applied through electrodes on the palms and plantar surfaces of the feet. All measurements were conducted in the morning. Participants were asked to urinate and defecate before the measurements and to avoid immediate postprandial assessment. Their palms and plantar surfaces were wiped with alcohol‐free wet wipes to clean and moisten them before stepping onto the electrode surface of the analyzer and grasping the hand electrodes. Body fat percentage (%BF) and FFM were analyzed based on measured body composition.

The BIA analyzer recorded Xc, R, and PhA along with body composition. PhA was calculated using Xc and R at 50 kHz AC (Ward & Brantlov, 2023) as the arctangent between the R and X; (X/R) × (180°/π). The mean PhA values for the right and left sides of the body at 50 kHz AC were used in the analysis and evaluated in absolute values.

### 2.3 Data analysis

Measurements were analyzed separately for males and females. Means and standard deviations were calculated for each group, and box‐and‐whisker plots were generated for body composition and PhA. The Kruskal–Wallis test was used to compare each measure between groups, and the Dunn–Šidák test was applied for multiple comparisons. In addition to the significance analyses, Cohen's *d* values were calculated as the effect size between the groups: *d* ≥ 0.8 are considered a large effect, ≤0.2 a small effect, and all other values a moderate effect. Pearson's correlation coefficients were calculated to assess the relationship between PhA and BMI in the Sports group, Normal group, and the overall sample. As mentioned in Section 2.1, data from high‐competition‐level students are included in the Appendix due to the small number of participants. The Mann–Whitney *U* test was used to compare body composition and PhA between this group and the L‐Sports group.

The StatFlex software (ver. 7.0.10; Artec, Osaka, Japan) was used for statistical analyses, with statistical significance set at *p* < 0.05.

## 3 RESULTS

**FIGURE 2:**
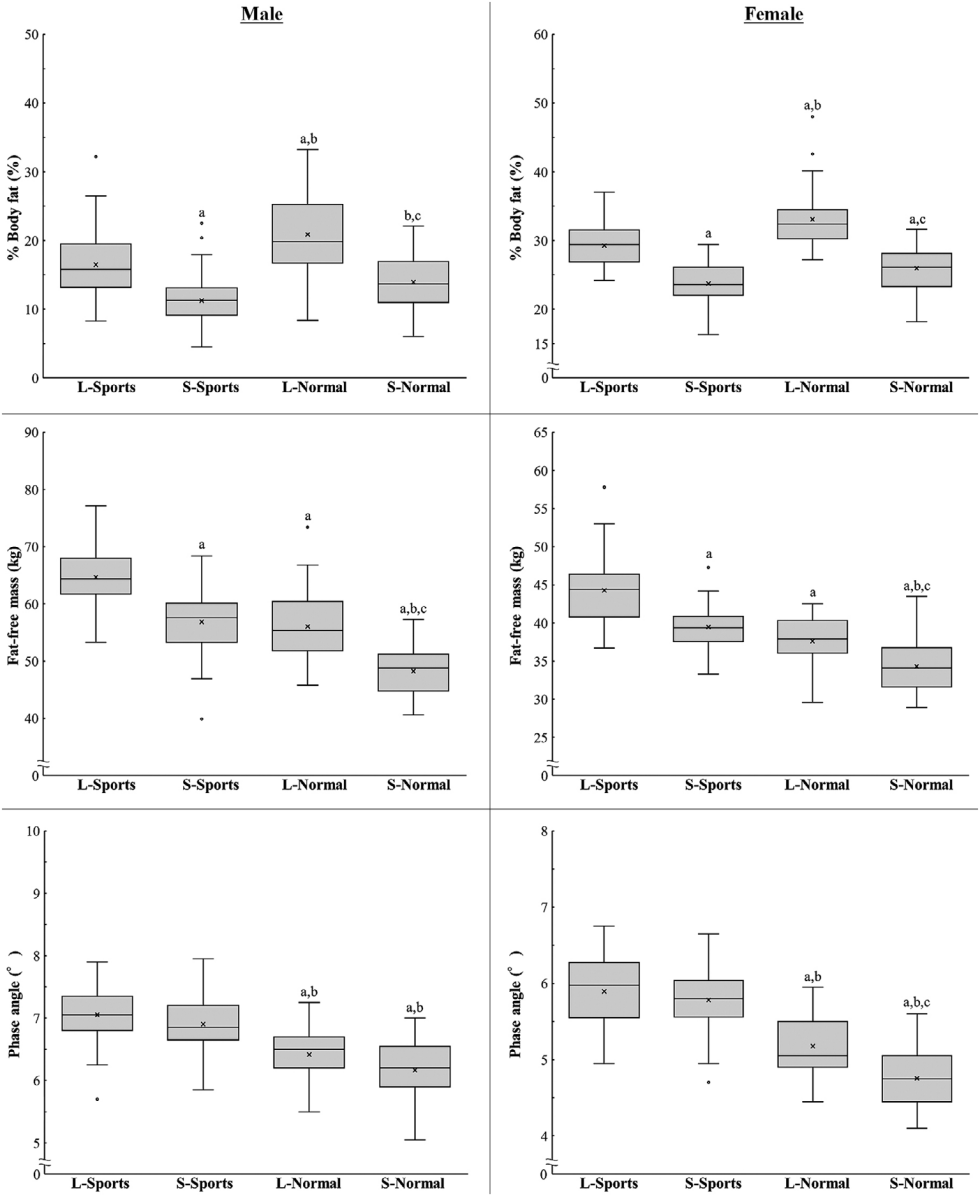
Box‐and‐whisker diagram of body fat percentage (top), fat‐free mass (middle), and phase angle (bottom) for each group. Alphabets indicate significant differences by multiple comparisons (*p* < 0.05; a vs. L‐Sports group, b vs. S‐Sports group, and c vs. L‐Normal group).

Table 1 presents the mean and standard deviation for each measure in each group, while Table 2 shows the effect sizes for comparisons between groups. Box‐and‐whisker plots for body composition and PhA are shown in Figure 2. For males: %BF (Figure 2, top left) was significantly higher in the L‐Normal group than in other groups and significantly lower in the S‐Sports group. FFM (Figure 2, middle left) was significantly higher in the L‐Sports group and significantly lower in the S‐Normal group, with no significant difference between the S‐Sports and L‐Normal groups. PhA (Figure 2, bottom left) was significantly higher in the Sports group than in the Normal group but did not differ between the Sports and Normal groups.

**TABLE 1:** Mean ± standard deviation of measured variables in each group.

	(a) L‐sports	(b) S‐sports	(c) L‐Normal	(d) S‐Normal	Kruskal–Wallis test	
					*H*	*p*
Male						
*N*	63	65	34	37		
Height (cm)	173.9 ± 5.6	172.1 ± 6.5	171.7 ± 4.7	170.1 ± 5.4	10.56	<0.05
Weight (kg)	77.7 ± 8.8	64.1 ± 6.1^a^	71.4 ± 10.6^a,b^	56.1 ± 4.9^a,b,c^	115.21	<0.01
BMI (kg/m^2^)	25.7 ± 2.6	21.6 ± 1.1^a^	24.2 ± 3.2^a,b^	19.4 ± 1.0^a,b,c^	146.46	<0.01
% Body fat (%)	16.5 ± 4.7	11.3 ± 3.4^a^	20.9 ± 5.9^a,b^	13.9 ± 4.3^b,c^	73.77	<0.01
Fat‐free mass (kg)	64.7 ± 5.4	56.8 ± 5.1^a^	56.1 ± 5.7^a^	48.2 ± 4.3^a,b,c^	112.54	<0.01
Phase angle (°)	7.05 ± 0.45	6.90 ± 0.46	6.41 ± 0.49^a,b^	6.17 ± 0.47^a,b^	71.68	<0.01
Female						
*N*	39	40	41	42		
Height (cm)	163.3 ± 6.3	160.0 ± 5.2	157.2 ± 5.7^a^	157.5 ± 6.5^a^	21.34	<0.01
Weight (kg)	62.7 ± 6.9	51.9 ± 4.1^a^	56.4 ± 6.2^a,b^	46.4 ± 5.1^a,b,c^	95.18	<0.01
BMI (kg/m^2^)	23.5 ± 1.4	20.2 ± 1.0^a^	22.9 ± 2.4^a,b^	18.7 ± 1.2^a,b,c^	121.72	<0.01
% Body fat (%)	29.3 ± 3.0	23.7 ± 3.1^a^	33.1 ± 4.1^a,b^	26.0 ± 3.2^a,b,c^	93.76	<0.01
Fat‐free mass (kg)	44.3 ± 4.5	39.5 ± 2.6^a^	37.6 ± 3.0^a^	34.3 ± 3.4^a,b,c^	86.75	<0.01
Phase angle (°)	5.88 ± 0.50	5.78 ± 0.43	5.18 ± 0.41^a,b^	4.75 ± 0.40^a,b,c^	89.11	<0.01

*Note*: Alphabets indicate significant differences by multiple comparisons (*p* < 0.05; a vs. L‐Sports group, b vs. S‐Sports group, and c vs. L‐Normal group).

**TABLE 2:** Effect sizes (*d*‐values) of measured variables between groups.

	L‐sports vs. S‐sports	L‐sports vs. L‐Normal	L‐sports vs. S‐Normal	S‐sports vs. L‐Normal	S‐sports vs. S‐Normal	L‐Normal vs. S‐Normal
Male						
Height	0.30	0.41	0.68	0.07	0.32	0.31
Weight	1.79	0.66	2.83	0.92	1.40	1.88
BMI	2.06	0.53	2.95	1.23	2.10	2.06
% Body fat	1.27	0.85	0.56	2.18	0.72	1.36
Fat‐free mass	1.49	1.56	3.27	0.15	1.78	1.55
Phase angle	0.33	1.37	1.92	1.04	1.59	0.52
Female						
Height	0.57	1.03	0.92	0.53	0.44	0.05
Weight	1.93	0.96	2.70	0.87	1.17	1.76
BMI	2.61	0.30	3.63	1.40	1.36	2.18
% Body fat	1.83	1.06	1.07	2.57	0.71	1.93
Fat‐free mass	1.32	1.75	2.53	0.66	1.73	1.03
Phase angle	0.22	1.55	2.49	1.44	2.46	1.05

For females: %BF (Figure 2, top right) was significantly higher in the L‐Normal group than in other groups and significantly lower in the S‐Sports and S‐Normal groups. FFM (Figure 2, middle right) was significantly higher in the L‐Sports group and significantly lower in the S‐Normal group, with no significant differences between the S‐Sports and the L‐Normal groups. The PhA (Figure 2, bottom right) was significantly higher in the Sports group than in the Normal group and significantly lower in the S‐Normal group than in the L‐Normal group. However, no significant differences were observed within the Sports group.

**FIGURE 3:**
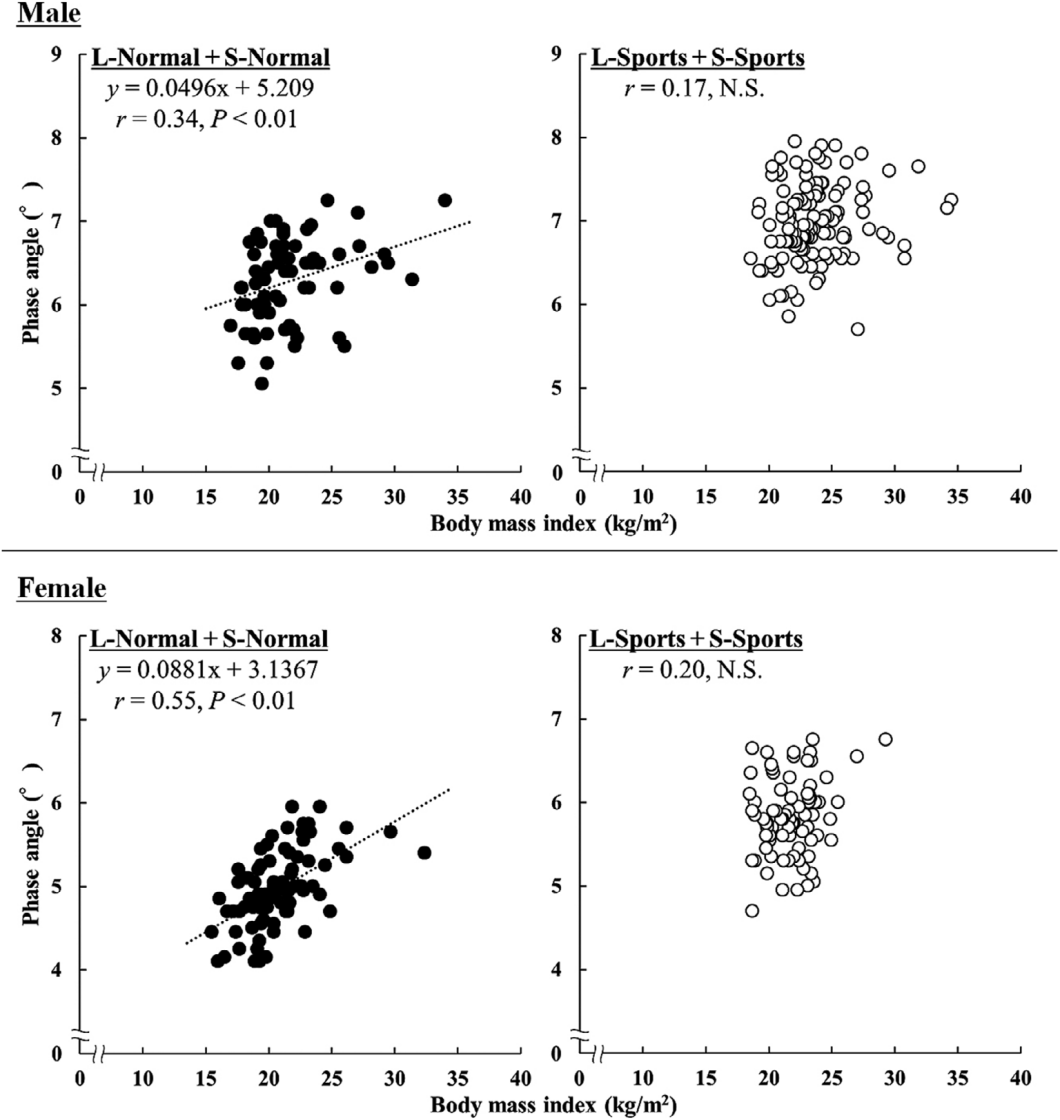
Relationship between phase angle and body mass index in nonathlete students (closed circle) and athletes (open circle).

Figure 3 shows the relationship between the PhA and BMI in the Sports and Normal groups. PhA was significantly and positively correlated with BMI in the Normal group, whereas no significant correlation was found in the Sports group. However, when analyzing all participants collectively, PhA and BMI showed a significant positive correlation (*r* = 0.36 and 0.43 for males and females, *p* < 0.01).

Means ± standard deviations (min–max values) for the higher competition group are presented in the Appendix. For males: %BF was 18.1 ± 4.1% (11.3–24.2%), FFM was 71.5 ± 7.0 kg (60.8–86.4 kg), and PhA was 7.3 ± 0.5° (6.1°–8.0°). %BF was not significantly different from that of the L‐Sports group (*p* = 0.11, *d* = 0.36), while FFM and PhA were significantly lower (*p* ≤ 0.01), with large (*d* = 1.17) and moderate (*d* = 0.57). For females: %BF was 31.6 ± 5.1% (24.4°–38.3%), FFM was 49.4 ± 3.5 kg (42.6–53.7 kg), and PhA was 6.5 ± 0.4° (5.7°–7.2°). %BF was not significantly different from that of the L‐Sports group (*p* = 0.61, *d* = 0.64), while FFM and PhA were significantly lower (*p* < 0.01), with large effect sizes (*d* = 1.19 and *d* = 1.29, respectively).

## 4 DISCUSSION

The mean PhA of the Normal group ranges from 6.2° to 6.4° in males and from 4.8° to 5.2° in females. PhA differs according to race (Barbosa‐Silva et al., 2005; Gonzalez et al., 2016; Zhang et al., 2024). For the same Japanese data as in the present study, the PhA is 6.22°–6.56° for males and 5.12°–5.37° for females aged 15–29 years (Oshita et al., 2025), and 6.3° for males and 5.4° for females aged 18–20 years (Akamatsu et al., 2022), which is approximately equivalent to our results. Although the FFM is not significantly different between the S‐Sports and L‐Normal groups, the PhA is higher in the S‐Sports group, with a large effect size. In contrast, although the FFM is higher in the L‐Sports group than in the S‐Sports group, the PhA is not significantly different, and the effect size is small. These results suggest that PhA is an indicator of differences in activity levels that are not reflected in the FFM. Previous studies have shown that the PhA is higher in athletes than in nonathletes and that its value is positively correlated not only with muscle mass but also with the intracellular/extracellular water ratio (Campa et al., 2021; Campa & Coratella, 2021; Lukaski & Raymond‐Pope, 2021). Furthermore, exercise training causes a significant increase in the intracellular compartment (Lukaski & Raymond‐Pope, 2021), and an increase in this compartment is associated with improvements in power and strength performance tasks, independent of weight and lean soft tissue changes (Silva, 2019). Therefore, the higher PhA observed in the Sports group compared to the Normal group may be due to the differences in training status, which influence the intracellular compartment. PhA has been proposed as a means to distinguish athletes and exercisers based on these characteristics (Campa & Coratella, 2021), and the present study supports this suggestion. If PhA is an indicator of whether an individual is an athlete or an exerciser, it could be used to accurately estimate body composition using the BIA method (Campa & Coratella, 2021). The BIA method assesses body composition using an estimation formula based on impedance values. These formulas differ depending on whether the user is classified as an athlete or non‐athlete, based on "self‐assessment.” Therefore, if PhA can objectively differentiate athletes from non‐athletes, it could guide the selection of appropriate estimation equations.

The difference in body physique within the Sports groups may be due to variations in sports disciplines. However, as this study does not investigate the specific sports disciplines of these groups, it remains unclear whether their differences are attributable to discipline‐specific factors. Previous studies have reported that PhA is slightly higher in explosive athletes than in endurance athletes (Campa et al., 2022), while some studies have found no significant differences in PhA between sports disciplines (Marra et al., 2021) or between resistance and aerobic exercisers aged 32–69 years (Yamada et al., 2022). Therefore, while physique varies with the type of exercise and sport, PhA may not vary equally with physique. This study suggests that PhA does not differ significantly among tier 2 athletes (McKay et al., 2022), even when their physiques or FFM vary.

Although this study found no significant association between the BMI and PhA in the Sports group, a significant positive association was observed in the Normal group. A possible reason for this difference may be related to the nutritional status. As BMI is commonly used to assess nutritional status (Elia, 2003; Serón‐Arbeloa et al., 2022) or the balance between energy intake and expenditure (Ministry of Health, Labour and Welfare, 2019) in the general population, a lower BMI in the Normal group is thought to be partly due to lower dietary intake. Underfeeding can lead to a decrease in body mass and associated changes in body composition; a decrease in both FFM and BF has been observed (Scalfi et al., 2002), while an increase in the ratio of extracellular to intracellular water has also been reported (Vaisman et al., 1988). As PhA has also been proposed as a potential indicator of nutritional status (Norman et al., 2012), PhA and BMI may have been positively correlated in the Normal group. However, most of these previous studies are related to malnutrition and anorexia nervosa. Whether PhA reflects the nutritional status of healthy adults, such as the Normal group, needs further detailed investigation. In contrast, athletes with lower BMI values may have higher FFM and lower %BF. As a study investigating PhA in underweight individuals reported that PhA was higher in ballet dancers (high physical activity) but lower in constitutionally lean and underfeeding individuals (Marra et al., 2019), PhA in athletes may also involve factors other than physique. While FFM does not differ significantly between the S‐Sports and L‐Normal groups, the S‐Sports group has a significantly lower BMI and %BF. Thus, the BMI in athletes may not be significantly related to PhA, due to both nutritional status and differences in body composition resulting from habitual training. However, an analysis of the relationship between PhA and BMI across all participants reveals a significant positive correlation, suggesting that this relationship is more likely to be observed in individuals with a broader range of athletic and activity levels. Although speculative, no correlation is observed within the Sports group. If athletes competing at higher levels were included, the range of athletic or activity levels might be widened, potentially revealing a correlation.

Although the number of students competing at higher levels is limited in this study, it is natural for the number of athletes to decrease as competition level increases. Consequently, data for athletes at or above the national level are presented in the Appendix and excluded from the primary analysis. The following discussion is for reference only. The participants included in the Appendix data were classified as tier 3 (national level) or tier 4 (international level) (McKay et al., 2022), but not as tier 5 (world class) (McKay et al., 2022). The FFM and PhA are significantly higher in this group, despite BMI being comparable to that of the L‐Sports group. However, the effect size in males is large for FFM and moderate for PhA. These results differ from a previous study, which found a greater increase in PhA than in FFM following resistance training (Lukaski & Raymond‐Pope, 2021), whereas in this study, greater differences are found in FFM at higher competition levels. Although this mechanism requires further investigation through longitudinal studies in different sports, the degree of increase in PhA diminishes at higher competition levels. In contrast, among females, both FFM and PhA (*d* = 1.19 and 1.29) show large effect sizes between groups, suggesting that PhA may be a better indicator of differences in competition levels in females. Future studies with larger samples of tier 3 and tier 4 athletes should explore these relationships further, considering differences in sports discipline. Further, if future studies demonstrate that PhA varies by tier classification or sports discipline, BIA‐based body composition estimation may need to be tailored to different levels or sports disciplines.

The limitations of this study are as follows. First, as a cross‐sectional study, it cannot establish causal relationships between PhA, body physique, and athletic/activity levels. Therefore, a longitudinal study is needed to investigate the changes in PhA and physique. In the context of sports research, whether PhA reflects differences in athletic levels may be of great interest. This study only examines differences in PhA between nonathlete students and trained/developing athletes. Future studies should explore how PhA varies across tiers 2–5 and among different sports disciplines. Finally, although BIA was performed with various controls in this study, as mentioned in 2.2, other considerations may have been necessary to improve the accuracy of the measurement. For example, menstruation is thought to have little or no effect on BIA measurement in females (Gualdi‐Russo & Toselli, 2002; McKee & Cameron, 1997). However, future studies may need to consider that PhA may be affected by menstruation. According to the instructions for use, measurement immediately after a meal was avoided in this study. However, a previous study suggested that ingestion of a meal or beverage may have an effect on Z, which may decrease over a period of 2‐4 h after a meal, generally representing a change of < 3% in Z values (Kushner et al., 2019). Therefore, future studies may need to control for postprandial time and the degree of dehydration caused by beverages. In addition, although the measurements were not conducted immediately after exercise and those with injuries that prevented them from exercising were not included, the previous day's exercise status and training period (in‐season, off‐season, etc.) were not investigated. Therefore, future studies will need to have a more detailed control of the measurement of the BIA.

## 5 CONCLUSION

This study investigated body physique, body composition, and PhA in nonathlete and trained/developmental‐level student athletes. A significant positive correlation was found between PhA and BMI only in nonathletic students, with larger physiques exhibiting a higher PhA. However, while FFM does not differ between students with larger physiques and athletes with smaller physiques, PhA is higher in the athletes. Although FFM differs by physique among athletes, PhA does not significantly vary. Furthermore, no significant relationship is found between PhA and BMI in athletes. These findings suggest that PhA serves as an indicator of differences in athletic/activity levels that are not captured by body physique or muscle mass. Therefore, PhA can be proposed as a means of distinguishing between athletes and exercisers which may help in selecting the estimating equation in the BIA.

## FUNDING INFORMATION

This work was supported by the Okayama Foundation for Science and Technology and JSPS KAKENHI (grant number JP24K09636).

## CONFLICT OF INTEREST STATEMENT

The authors declare that they have no competing interests.

## ETHICS STATEMENT

All participants provided written informed consent before enrolment in the present study, which was approved by the Research Ethics Committee of Kyushu Kyoritsu University (2022–08).

## Data Availability

The datasets generated and analyzed in the current study are available from the corresponding author upon reasonable request.

## Appendix

Comparison of body composition and PhA between the L‐Sports group and a higher competition (national/international level) group with the same BMI range.
